# Book Reviews

**Published:** 1799-04

**Authors:** 


### MEDICINE AND SURGERY.

*The effect of the Nitrous Vapour, in preventing and deſtroying Contagion, aſcertained from a variety of trials, made chiefly by Surgeons of His Majeſty's Navy, in Priſons, Hoſpitals and on board of Ships: with an Introduction reſpecting the nature of the Contagion which gives riſe to the Jail or Hoſpital Fever; and the various methods formerly employed to prevent or deſtroy this:* By James Carmichael Smith. M. D. F. R. S. Fellow of the Royal College of Phyſicians, and Phyſician Extraordinary to his Majeſty. 8vo. 231. pp. *London*; Johnſon. Price 5s.

In an explanatory preface, the author informs us that the experiments made with the nitrous fumigation, during the laſt three years, or ſince the time when he publiſhed an account of it, have proved, in the cleareſt and moſt unequivocal manner, to every unbiaſſed mind, not only the power of the nitrous vapour in deſtroying contagion, but the ſafety with which it might be uſed.

The introduction, from page 1 to 48, contains thoſe general obſervations on the jail and other putrid contagions, with the uſual means of obviating theſe, formerly publiſhed in the '*Treatiſe on the Fever at Wincheſter,*' and now republiſhed, from a wiſh to make them more generally known. The principal part of this work conſiſts, 1, of a '*Report of the Experiment for ſtopping the Progreſs of Contagion, as executed on board the Union Hoſpital-ſhip, at Sheerneſs; by Mr. Arch. Menzies:*' from page 49 to 114.—and 2, of a Collection of Letters by Meſſrs. *Paterſon*, *M'Grigor*, *Hill*, *Griffin*, *Glegg*, *Magennis*, *Snipe*, *Blatherwick*, *Brown*, and *Drew*, ſurgeons of different ſhips and hoſpitals; a letter from Capt. *Lane*, Farham; extracts from Letters or Journals of Surgeons of the Navy, tranſmitted to the Board of Sick and Hurt; and the correſpondence between Drs. *Withering*, *Duncan*, *Percival*, and the Authors relative to this ſubject.

*Memoirs of the Yellow Fever, which prevailed in Philadelphia, and other parts of the United States of America, in the Summer and Autumu of the preſent Year,* 1798; *including Tables of the Weather, &c.*" By William Currie, S. C. M. P. author of an Hiſtorical Account of the Climates and Diſeaſes of the United States, &c. &c. 8vo. 145 pp. *Philadelphia.* Dobſon.

This pamphlet contains a collection of facts truly valuable to every medical practitioner, but the author has been at no pains to arrange them. He

He begins his work with a sketch of the weather from January to July, 1798, and opens this melancholy budget of disease and death, with the first of August, when the first case of yellow fever occurred, which proved fatal on the 4th. He then proceeds, in chronological order, with detailing new cases, addresses to the public by magistrates and physicians, new methods of treatment, number of deaths, and occasionally too, accounts of robberies committed, &c. The month of September is likewise prefaced with a Table of the Weather, together with daily returns of the sick and the deaths in that month. A gloomy picture indeed! as, in the city of Philadelphia alone, not less than 3446 persons fell victims to this malignant epidemic, between the 1st of August and 3d of November!

Dr. Currie endeavours to prove, by a great number of concurrent circumstances, that the contagion, which gave origin to the late epidemic, was imported in the ship Deborah, which arrived at Philadelphia, from Jeremie, on the 18th of July last. He further quotes the opinion of the College of Physicians of that city, who agree with him, that the yellow fever is derived from imported contagion, and who, for this opinion, assign the following, among other reasons:

"The disease in question 'say the college' is essentially different from the fevers that occur in this climate, and which originate from domestic causes. It also differs from them essentially, in the circumstance of being contagious; a bilious fever originating from domestic causes, having never been to our knowledge contagious in this climate."

*An Outline of the History and Cure of Fever, endemic and contagious; more expressly the contagious Fever of Jails, Ships, and Hospitals; the concentrated endemic, vulgarly called the Yellow Fever of the West Indies: To which is added, an explanation of the principles of military discipline and economy; with a scheme of medical arrangements for armies.* By ROBERT JACKSON, M. D. 8vo. 396 pp *Edinburg*, Mundell and Son; *London*, Longman, Murray and Highley. Price Seven Shillings.

In an advertisement prefixed to this work, the author modestly mentions the opportunities he has had of making medical observations, since the year 1774, as an army surgeon, first in Jamaica, and afterwards in America, where he had opportunities of observing very extensively the different forms and degrees of fever, in the southern states. In the year 1795, he was again ordered to the West Indies—to the island of St. Domingo, where the duty assigned to him afforded the means of examining the appearance of things, at different posts, and in different districts, more fully than fell to the lot of any other person on the medical staff of that island. As a considerable portion of this work consists of valuable and interesting matter, together with many original remarks and observations, we propose to furnish our readers with a concise abstract of its practical information, in a future number; as the room allotted to this 'Retrospect' does not admit of extensive quotations.

